# Biological Functions and Cross-Kingdom Host Gene Regulation of Small RNAs in *Lactobacillus plantarum*-Derived Extracellular Vesicles

**DOI:** 10.3389/fmicb.2022.944361

**Published:** 2022-08-18

**Authors:** Siran Yu, Zhehao Zhao, Piliang Hao, Yan Qiu, Meiyi Zhao, Gang Zhou, Chengqian Zhang, Jiuhong Kang, Ping Li

**Affiliations:** ^1^Clinical and Translational Research Center of Shanghai First Maternity and Infant Hospital, School of Life Sciences and Technology, Tongji University, Shanghai, China; ^2^School of Life Science and Technology, ShanghaiTech University, Shanghai, China

**Keywords:** microRNA, microRNA-size RNA (msRNA), *lactobacillus*-derived extracellular vesicles (LDEVs), post-transcriptional regulation, microbe-host interspecies communication

## Abstract

Extracellular vesicle-mediated transfer of microRNAs is a novel mode of cell-to-cell genetic transmission. Extracellular vesicles produced by microbes have been shown to contain significant quantities of physiologically active molecules such as proteins, lipids, and RNA, which could be transported to host cells and play a key role in both inter-kingdom signaling and physiological responses. In this study, we identified sRNAs by sequencing small RNAs (sRNAs) from *Lactobacillus plantarum*-derived extracellular vesicles (LDEVs) and detected the expression levels of vesicular sRNAs using quantitative reverse transcription-polymerase chain reaction (RT-PCR), which demonstrated the presence of microRNA-sized RNAs (msRNAs) within these vesicles. We chose sRNA71, a highly expressed msRNA, for further investigation, predicted its potential target genes for the human genome, and indicated that it could be translocated into mammalian cells. The biological functions of this sRNA71 were subsequently explored through cellular proteomics, western blot, and luciferase reporter assay. According to the findings, transfection with synthetic sRNA71 mimics substantially reduced Tp53 expression in HEK293T cells and suppressed the gene expression through binding to the 3′ UTR of Tp53 mRNA. In conclusion, it is hypothesized that microbial-derived extracellular vesicles serve as carriers of functional molecules such as sRNAs, which play an essential role in regulating microbial-host communication.

## Introduction

Bacterial extracellular vesicles (EVs), also known as outer membrane vesicles (OMVs), are naturally generated by all gram-negative bacteria and feature nano-sized (20–250 nm) lipid-bilayered vesicular structures that contain a variety of immunostimulatory components (Kulp and Kuehn, 2010; Brown et al., 2015; Kim et al., 2015). The phenomenon of OMVs produced by gram-negative bacteria has been observed since 1966. Until 2009, Lee et al. (2009) isolated MVs shed by gram-positive bacteria from the culture supernatants of *Staphylococcus aureus* (*S. aureus*) and *Bacillus subtilis*. According to current research, these bacterial EVs serve crucial physiological and pathological roles as potential mediators in bacterial-bacterial and bacterial-host interactions (Ahmadi et al., 2017; Lefebvre and Lecuyer, 2017).

*Lactobacilli* are beneficial components of the human intestinal microbiota and constitute a stable, moderately rich, and biodiverse community in the intestinal microbiota (Rossi et al., 2016). *Lactobacillus* is believed to be functionally dominant, for example, enhancing intestinal barrier function (Wang et al., 2018) and improving the body’s physiological processes and cognitive capacities through controlling gut flora (Ni et al., 2019). The biological activities of *Lactobacillus plantarum* (*L. plantarum*)-derived extracellular vesicles (LDEVs) have attracted the attention of scientists due to the important role played by LDEVs in immunomodulation of colon cancer. Li et al. (2017) reported that LDEVs could be transported into human colon cells Caco-2, upregulated the expressions of host defense genes *CTSB* and *REG3G* in Caco-2 after LDEVs uptake, and enhanced host immune responses against vancomycin-resistant *enterococci*. Furthermore, Kim et al. (2018) revealed that LDEVs protected atopic dermatitis induced by *S. aureus*-derived EVs. Recently, it was shown that LDEVs enhance BDNF expression in cultured hippocampal neurons and produce antidepressant-like effects in mice (Choi et al., 2019). These results demonstrate that LDEVs play significant biological roles in interspecies communication and signaling events, but the particular underlying mechanisms need to be investigated further since the active functional components in LDEVs have not been thoroughly proven. Exploring the components of LDEVs that have biological activity and studying their possible functions is critical for understanding the interaction between microbe and the host.

There is mounting evidence that microRNAs (miRNAs) enclosed in mammalian-derived exosomes might operate as important gene regulatory factors, which made exosomes become the novel mediators of genetic exchanges between mammalian cells (Valadi et al., 2007). As a remarkable class of small regulatory non-coding RNAs (sRNAs) with about 17–25 nucleotides (nt) in length, miRNAs could perform the ability of RNA interference (RNAi) and post-transcriptional regulation to silence gene expression by complementary base pairing with the 3′ untranslated region (UTR) of target mRNA after incorporating into the RNA-induced silencing complex (RISC) (Bartel, 2004; Eulalio et al., 2008). Resembling exosomes, bacterial vesicles also contain RNAs (Tsatsaronis et al., 2018). As early as 1989, DNAs and RNAs were detected in bacterial membrane vesicles (Dorward et al., 1989). In 2014, Biller et al. (2014) employed deep RNA sequencing to reveal the diversity of RNAs in EVs shed by the marine cyanobacterium *Prochlorococcus*. Following analysis of the RNA content and class of EVs from gram-negative (Ghosal et al., 2015; Koeppen et al., 2016; Choi et al., 2017; Malabirade et al., 2018) and gram-positive (Bitto et al., 2021; Luz et al., 2021) bacterial species, it seems that the packaging of extracellular RNAs, predominantly sRNAs < 100 nt in length, in EVs is a widespread phenomenon. Accompanied with research evidence increasing progressively, functional investigations of sRNAs carried by bacterial MVs have gradually been conducted. Only sRNAs within gram-negative bacteria OMVs were mentioned to represent the potential to cross-species regulate host gene expression. Koeppen et al. (2016) performed small RNA sequencing on OMVs released by *Pseudomonas aeruginosa* (*P. aeruginosa*), predicted that vesicular sRNAs could potentially target human immune genes, and demonstrated for the first time that sRNA52320 decreased the expressions of MAP2K4 and MAP3K7 in LPS-induced MAPK signaling pathways. Moreover, Choi et al. (2017) identified the presence of miRNA-size RNAs (msRNAs) in OMVs derived from *Aggregatibacter actinomycetemcomitans*, *Porphyromonas gingivalis*, and *Treponema denticola*, such as A.A_20050, P.G_45033, and T.D_2161, respectively. MicroRNA-like molecules secreted from these periodontal pathogens *via* bacterial OMVs could be delivered to fibroblast NIH3T3. The levels of cytokines IL-5, IL-13, and IL-15 in Jurkat T cells were depressed after transfection of synthetic msRNA oligos. Studies mentioned above indicated that sRNAs contained within bacterial EVs might act functionally analogous to miRNAs enclosed in mammalian-derived exosomes and that they can modulate host gene expressions, potentially implicated in interspecies communication. We previously showed that EVs promoted the growth of *L. plantarum* WCFS1, which is aided by enabling its vector to modify bacterial genes (Yu et al., 2019). Since the potential functions of EV-associated cargos, the effects of LDEVs *in vitro* have been investigated. The RNA profile of LDEVs, on the other hand, has not been well studied. The major chemical components of LDEVs in terms of biological roles in controlling gene expression remain unknown. As a result, further study into extracellular RNAs in LDEVs is required to better understand their biological involvement in microbe-host interactions.

The purpose of this study was to evaluate the biological functions of sRNAs in LDEVs as well as the underlying mechanisms by which they perform in their host role. LDEVs were isolated from the culture supernatant of *L. plantarum* WCFS1 using ultracentrifugation. Small RNA sequencing (RNA-seq) was performed, and sRNA expression levels were determined using reverse transcription-polymerase chain reaction (RT-PCR). Bioinformatics analysis was applied for the target prediction of bacterial sRNAs and the associated signaling pathways. One highly expressed sequence (sRNA71) was selected and synthesized for further study. After cell transfection of sRNA71 mimics or negative control in HEK293T cells, proteomics, western blot, and luciferase assay were conducted to reveal the role of sRNA71 in host cell biology. Our research showed that sRNA71 could employ well-established mechanisms similar to those of miRNAs to inhibit Tp53 by short seed pairing within the 3′ UTR in interspecies communication.

## Results

### Detection of Small RNAs in *Lactobacillus plantarum*-Derived Extracellular Vesicles

**FIGURE 1:**
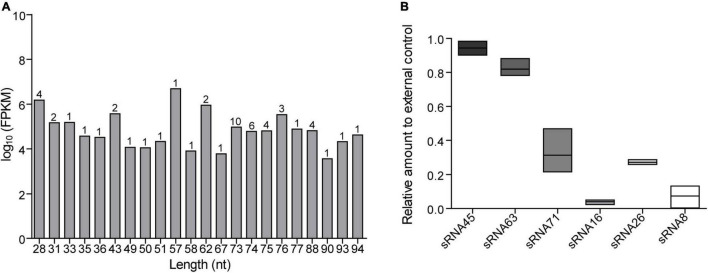
RNA sequencing results and relative expression levels of sRNAs in LDEVs. **(A)** Length distribution of sequenced sRNAs. The numbers on the columns represent the number of sRNAs of the same length. **(B)** The relative amount of each sRNA was determined *via* RT-PCR. About 1 pmol external control and standard curve were used for calculation. Data were shown as mean ± SEM (*n* = 3 biological replicates). The results were presented using GraphPad Prism 5.00.

We isolated and characterized LDEVs from *L. plantarum* WCFS1 by a published procedure (Yu et al., 2019; Supplementary Figure 1A). LDEVs ranged from 37.8 to 459 nm in size (Supplementary Figure 1B), which is consistent with the size range of bacterial MVs. RNAs harboring LDEVs were extracted after the isolation of vesicles derived from bacterial culture supernatants. RNA quality was analyzed by a bioanalyzer (Supplementary Figure 1C) before the preparation of cDNA libraries. RNA integrity value (RIN) was 1.80, which was enough for sequencing requirements. Through data trimming and filtering, a total of 2,765,069,535 sequence reads was obtained. After sequencing analysis, we identified 52 abundant sRNA sequences in LDEVs purified from the supernatants of WCFS1 (Supplementary Table 1), predominantly sRNAs < 100 nt in length, which contains 29 transfer RNA (tRNA) fragments and 23 unknown predicted sequences. The top 10 sRNAs with a length less than 100 nt were listed in Table 1. The fragments per kilobases of exon per million fragments mapped (FPKM) of each sRNA in the sample were representative of its expression quantity. The sequence length distribution of 52 sRNAs was shown in Figure 1A. Based on the results of RNA-seq, there are four sequences that we refer to as microRNA-like molecules or msRNAs due to the length being similar to those of classical eukaryotic miRNAs. Reads of predicted sequences in LDEVs account for 91.5% among 52 sRNAs < 100 nt in length. Using the Vienna RNA package RNAfold software, the secondary structure of predicted sequences was predicted and described in Supplementary Figure 2. Bioinformatics approaches showed that abundant sRNAs contained within LDEVs such as sRNA45 and sRNA63 were predicted to form stable secondary structures very similar to those of precursor miRNAs, which indicates that LDEV-derived sRNAs play a role in the regulation of host mRNA. Since bacterial small RNAs might mimic eukaryotic microRNAs targeting the host, sRNAs identified in LDEVs may function through an RNAi mechanism by pairing with complementary target genes. We focused on the roles of sRNAs and their abilities to interact with the host to reveal the biological activities of LDEVs. To validate the presence of sRNAs, as well as the results of RNA-seq, the relative expression levels of sRNAs in LDEVs were measured (Figure 1B). Standard curves of each sRNA and external control for miRNAs were illustrated in Supplementary Figure 3. Consistent with the results of RNA-seq, sRNA45 was the highest abundant in LDEVs. The FPKM of sRNA16 was greater than that of sRNA26 (Table 1), which indicated that the content of sRNA16 in LDEVs was higher. Conversely, the relative amount of sRNA16 was lower than that of sRNA26 obtained by RT-PCR, which was almost like sRNA71 (Figure 1B). We proposed that more than one method for the determination of RNA contents was essential.

**TABLE 1:** Top 10 most prevalent sRNA reads of LDEVs.

RNA name	Length (nt)	FPKM	Sequence
sRNA45	57	4,888,407	GTTTCCCAGTTTCCGATGCACTTCTTCGGTTGAGCCGAAGGCTTTCACATCAGACTT
sRNA63	62	868,243	CTCAAGTTTCCTAGTTTCCGATGCACTTCTTCGGTTGAGCCGAAGGCTTTCACATCAGACTT
sRNA71	28	757,483.1	GTGCAAGAGCTTTCTTGTAATTTACGTG
sRNA16	28	378,741.6	TATTGAAATCGGAGAGACGTAATTATGC
sRNA56	43	352,533.7	GCCCCCGTCAATTCCTTTGAGTTTCAGCCTTGCGGCCGTACTC
sRNA26	28	189,370.8	CACAAACTCAATTTTTAATCTTAACTCG
sRNA8	28	189,369.9	AGTAGTAAATGGAATAGAATGGAGTAAT
sRNA31	31	73,801.4	CTAGGGCTATGCTGGTAAAGACGGGTATTTG
sRNA69	31	73,801.4	CCCAAAAGTATGACTGTGACTTATATAGATG
sRNA2	33	50313.39	GCGCTGAAACGCGTGACGATTAGACGTGTATTG

### *Lactobacillus plantarum*-Derived Extracellular Vesicle-Associated Sequencing Small RNAs Were Predicted to Target Human Genes

**FIGURE 2:**
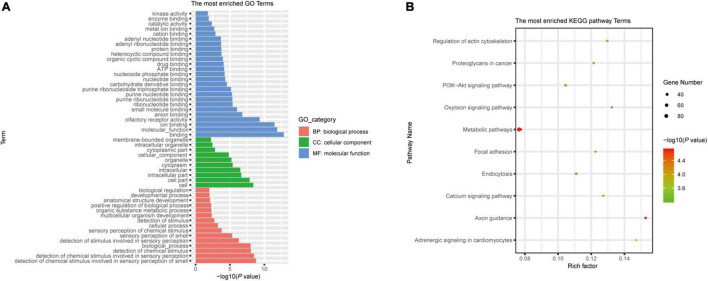
Bioinformatics analysis of predicted target genes of LDEVs-associated sRNA71. The graphs were made with R package “ggplot2” (version 3.6.1). **(A)** Top 50 correlative GO terms of potential target genes of sRNA71. Selected terms were divided into three major categories, including biological process, cellular component, and molecular function. **(B)** The most enriched KEGG pathways of candidate genes were targeted by sRNA71 (*P* < 0.001). Counting the number of candidate genes was served as “Gene Number,” which divided by total number of genes in this pathway equals “Rich factor.”

According to previously published predicted strategies (Koeppen et al., 2016; Zhao et al., 2018), potential LDEV-associated sRNAs target genes were accomplished in the human genome using the miRanda software. When the scoring threshold was 160, a total of 69,074 mRNA sequences were identified, representing 8,727 genes. Applying the PANTHER classification system^1^ for Gene Ontology (GO) annotation, an enrichment of predicted target genes was provided according to the biological process in which they participate, the molecular function, and the cellular component (Supplementary Figure 4A). Supplementary Figure 4B showed the results of the Kyoto Encyclopedia of Genes and Genomes (KEGG) pathway enrichment analysis. There was a total of 214 terms with *P* < 0.05. The most enriched GO and KEGG pathway terms were listed. A lower *P* indicates a higher statistical significance of this term. Based on the results of RNA-seq, we chose sRNA71 as a candidate for further analysis. There is a total of 1,754 predicted targets (Supplementary Table 2). GO analysis of those candidates revealed the significant enrichment of 50 terms (Figure 2A). A total of 1,754 targets were successfully recognized as members of 279 pathways (Supplementary Table 3), with 67 enriched KEGG pathways (*P* < 0.05). As shown in Figure 2B, the top five most enriched KEGG pathways were Axon guidance (hsa04360), Metabolic pathways (hsa01100), Regulation of actin cytoskeleton (hsa04810), Adrenergic signaling in cardiomyocytes (hsa04361), and PI3K-Akt signaling pathway (hsa04151). It revealed that sRNA71 potentially targets 36 genes in the PI3K-Akt signaling pathway, including *PI3K* (Entrez Gene ID: 5291, 5295), *AMPK* (Entrez Gene ID: 5562), *SGK* (Entrez Gene ID: 6446), *CREB* (Entrez Gene ID: 84699), and *p53* (Entrez Gene ID: 7157). Collectively, we speculated that extracellular sRNAs in LDEVs secreted from *L. plantarum* WCFS1, such as sRNA71, promisingly mediate host gene regulation in interspecies communication.

### Cellular Protein Expressions Were Modified by sRNA71

To determine our hypothesis, we attempted to study the function of sRNA71. Having detected the presence of sRNAs, we first determined whether sRNAs could be conveyed by LDEVs into mammalian cells. Consistent with previous studies, extracellular sRNAs transferred into mammalian cells *via* LDEVs were observed (Supplementary Figure 5). We stained LDEVs with lipophilic dye DiI to observe the localization inside the cytoplasm. In line with previous research, bacterial EVs could be internalized by mammalian cells (Canas et al., 2016; Li et al., 2017; Seo et al., 2018). As illustrated in Supplementary Figure 5C, co-culturing LDEVs with HEK293T cells increased the expression level of sRNA71, indicating that LDEVs fuse with mammalian cells releasing their cargo into host cells.

**FIGURE 3:**
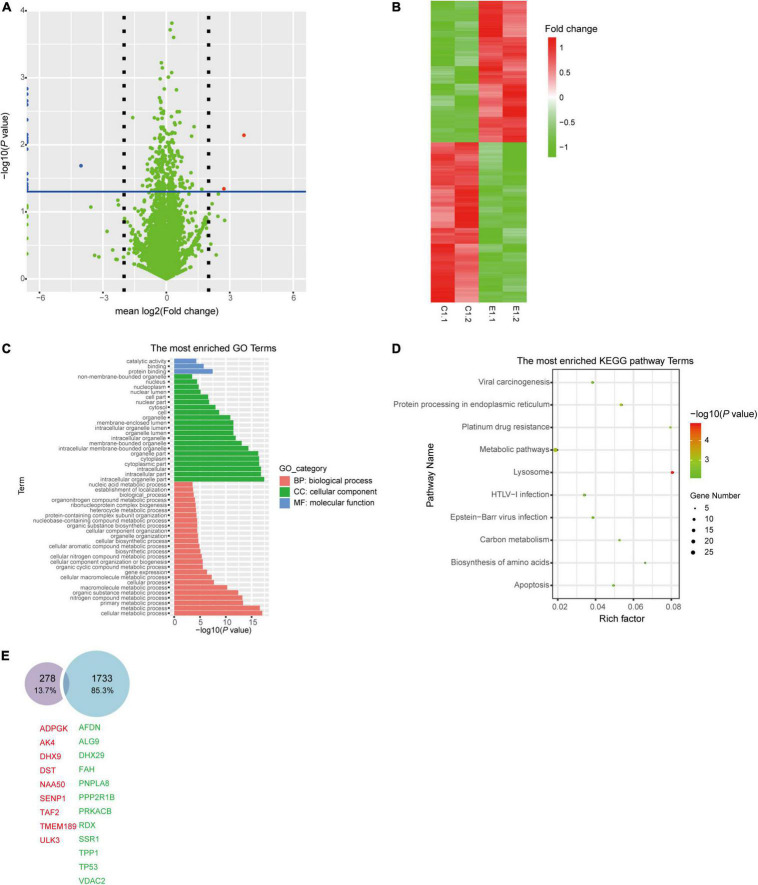
The abundance of cellular proteins were altered by sRNA71. **(A)** Volcano plot of −log_10_ *P*-values and mean log_2_ Fold change for 6,600 proteins from HEK293T cells transfected with sRNA71 mimics compared to those transfected with negative control. Red dot, upregulated proteins; Blue dot, downregulated proteins. **(B)** Proteomics heat map represented 309 dysregulated proteins in HEK293T cells which were determined by Student’s *t*-test (*P* < 0.05). All procedures in experimental groups (E1.1, E1.2, for short) were in accordance with control groups (C1.1, C1.2, for short). **(C,D)** GO annotation and KEGG pathway enrichment of 309 dysregulated proteins. The graphs were made with R package “ggplot2” (version 3.6.1). **(E)** Venn diagram depicts the number of the intersection between sRNA71 candidate target genes (blue circle) and genes corresponding to differentially expressed proteins (purple circle), including nine upregulated genes (red tag) and 12 downregulated genes (green tag).

To evaluate the correlation between bacterial sRNA with host genes and its impact on host signaling, cellular proteomics was performed. Before proteomics, synthetic sRNA71 mimics or its negative control was transfected into HEK293T cells for 48 h. Experiments with two biological replicates yielded 6,600 quantifiable proteins (Figure 3A). Related to the negative control, 309 protein expressions were significantly changed with a statistical difference (*P* < 0.05). The heat map revealed that transfection of sRNA71 mimics caused upregulated expression of 145 proteins and downregulated expression of 164 proteins (Figure 3B and Supplementary Table 4). Figures 3C,D exhibited the results of GO and KEGG pathway enrichment analysis of differentially expressed proteins, respectively. Among the biological processes, differentially expressed genes (DEGs) are distributed in several metabolic processes. We identified a total of 31 pathways regulated by sRNA71 mimics (*P* < 0.05). As illustrated in Figure 3D, genes corresponding to differential expression proteins were predicted to mainly participate in Lysosome (hsa04142), Protein processing in endoplasmic reticulum (hsa04141), Metabolic pathways (hsa01100), Platinum drug resistance (hsa01524), and Apoptosis (hsa04210). In particular, sRNA71 downregulated seven proteins such as FADD (Entrez Gene ID: 8772), Tp53 (Entrez Gene ID: 7157), and BAX (Entrez Gene ID: 581) which are connected to apoptosis. The predicted target genes of sRNA71 were co-analyzed with 299 genes corresponding to differentially expressed proteins. A Venn diagram showed an overlap with 21 genes (Figure 3E and Table 2), in which 12 genes were inhibited, suggesting the possibility of host gene regulation by sRNA71. We hypothesized that sRNA71 contained inside LDEVs secreted by *L. plantarum* WCFS1 used the comparable mechanism to exosome-borne miRNAs to affect host cell processes in interspecies interactions.

**TABLE 2:** Protein expressions of predicted target genes were upregulated or downregulated after the transfection of sRNA71 mimics.

Gene name	Protein description	Fold change^a^	Log_2_ (Fold change)	*P*-value	Regulation
ADPGK	ADP-dependent glucokinase	1.17	0.22	0.040	Up
AK4	Adenylate kinase 4	1.12	0.17	0.048	Up
DHX9	ATP-dependent RNA helicase A	1.11	0.14	0.041	Up
DST	Dystonin	1.26	0.34	0.027	Up
NAA50	N-alpha-acetyltransferase 50	1.05	0.08	0.027	Up
SENP1	Sentrin-specific protease 1	1.67	0.76	0.015	Up
TAF2	Transcription initiation factor TFIID subunit 2	1.10	0.14	0.044	Up
TMEM189	Transmembrane protein 189	6.60	2.72	0.045	Up
ULK3	Threonine-protein kinase	1.19	0.25	0.012	Up
AFDN	Afadin	0.90	-0.16	0.012	Down
ALG9	Alpha-1,2-mannosyltransferase	0.62	-0.70	0.034	Down
DHX29	ATP-dependent RNA helicase	0.89	-0.16	0.008	Down
FAH	Fumarylacetoace-tase	0.81	-0.30	0.008	Down
PNPLA8	Calcium-independent phospholipase A2-gamma	0.54	-0.88	0.023	Down
PPP2R1B	Threonine-protein phosphatase 2A 65 kDa regulatory subunit A beta isoform	0.84	-0.25	0.047	Down
PRKACB	cAMP-dependent protein kinase catalytic subunit beta	0.00	-∞^b^	0.043	Down
RDX	Radixin	0.79	-0.33	0.011	Down
SSR1	Translocon-associated protein subunit alpha	0.67	-0.57	0.004	Down
TPP1	Tripeptidyl-peptidase 1	0.78	-0.36	0.046	Down
TP53	Cellular tumor antigen p53	0.84	-0.25	0.005	Down
VDAC2	Voltage-dependent anion-selective channel protein 2 Gene	0.86	-0.21	0.022	Down

*^a^Fold change is calculated by dividing the protein intensity of the experimental group by that of the control group.*

*^b^Due to the expression of PRKACB was completely blocked by sRNA71 mimics compared with the negative control.*

### *Lactobacillus plantarum*-Derived Extracellular Vesicles-Derived sRNA71 Could Downregulate Tp53 in HEK293T Cells

**FIGURE 4:**
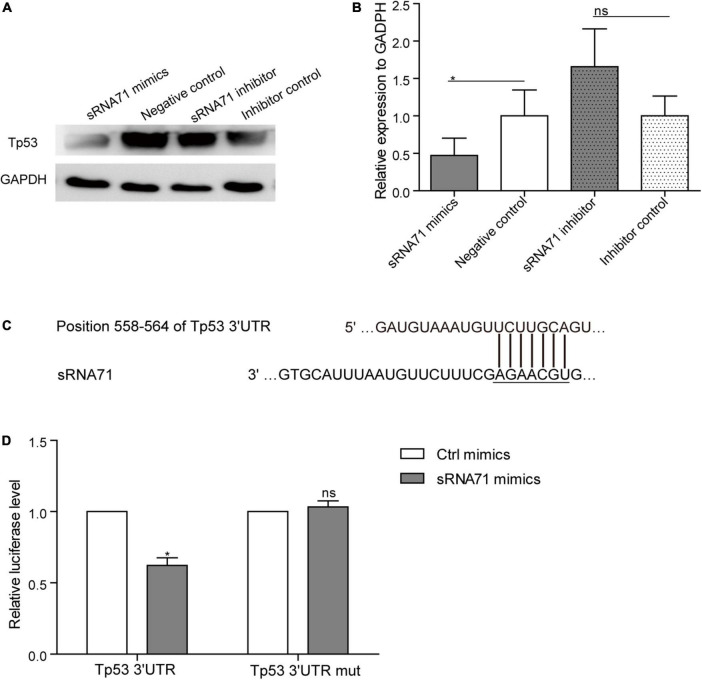
sRNA71 inhibited the expression of Tp53. **(A,B)** Western blot analysis of the expression of Tp53 in HEK293T cells transfected with sRNA71 mimics, negative control, sRNA71 inhibitor, or inhibitor control, respectively. Images are representative results of three independent experiments. **(B)** The protein abundance of Tp53 was quantified with normalization by signals of GAPDH. Means ± SEM from *n* = 3 independent experiments. **(C)** Schematic representation of the predicted binding site. **(D)** HEK293T cells transfected with Tp53 3′ UTR firefly luciferase reporter vector (Wild-type vector or mutant vector), renilla luciferase vector, and sRNA71 mimics were compared to those transfected with negative control for the same period of time. After 48 h, cells were lysed and monitored for luciferase activity. The relative luciferase level was normalized by the reference “Renilla.” Means ± SEM from *n* = 3 independent luciferase reporter assays, *P < 0.05 vs. negative control, “ns” reflects no statistically significant difference.

Based on proteomics data, approximately 0.2 times decrease of Tp53 in HEK293T cells after transfection of sRNA71 mimics for 48 h (Table 2). To verify the results, a western blot was conducted and revealed that sRNA71 mimics resulted in an obvious suppression of Tp53 (Figures 4A,B). We presumed that bacterial sRNAs might function like eukaryotic miRNAs, which repress translation by binding to the 3′ UTR of target mRNA (Figure 4C). To test this hypothesis, sRNA71 mimics or negative control were transfected in the presence of the reporter plasmids into HEK293T cells, and then the luciferase activity was detected. Related to the negative control, the luciferase level was reduced to 62.18% (Figure 4D). sRNA71 mimics markedly decreased the luciferase activity but barely affected that of the mutant vector. These results suggest that sRNA71 might bind to the 3′ UTR of Tp53, hence leading to the downregulation of Tp53. Overall, these findings are consistent with the hypothesis that host gene regulation by sRNA in microbe-host interactions.

## Discussion

Acting like miRNA from mammalian cells, microbial sRNA from an organism is a novel pattern of microbe-host communication that regulates the immune responses (Tsatsaronis et al., 2018; Lee, 2019), for instance, between fungi and plants. A bidirectional cross-kingdom RNAi mechanism in fungal pathogens *Botrytis cinerea* and *Arabidopsis thaliana* (Weiberg et al., 2013; Cai et al., 2018) were demonstrated. In line with Weiberg et al. (2013) and Wang et al. (2017) reported that *Puccinia striiformis* f. sp. *tritici* (*Pst*) microRNA-like RNA 1 (Pst-milR1) repressed the plant’s innate immunity by suppressing the expression of wheat pathogenesis-related 2, suggesting that Pst-milR1 might participate in cross-kingdom RNAi events. In terms of pathogen-host interaction, bacterial sRNAs were important for their pathogenicity and contributed to attenuating the host immune responses. In infected macrophage THP-1 cells, *Mycobacterium marinum* expressed MM-H that could be bound by RISC, implying its potential to silence host gene expression (Furuse et al., 2014). Westermann et al. (2016) applied dual RNA sequencing to identify the host cell-internalized *Salmonella enterica serovar Typhimurium* sRNA (namely PinT) and performed bioinformatics analysis to investigate the impacts of PinT on the JAK-STAT signaling pathway in Hela cells. Taken together, these results indicated the cross-kingdom regulation of host genes by microbial sRNAs.

There is emerging evidence that the majority of RNAs in microbial EVs are shorter than 250 nt in length (Tsatsaronis et al., 2018) and were therefore classified as sRNAs that comprise multiple different RNA biotypes, including miRNAs and others. In this paper, the RNA profile of *L. plantarum* WCFS1-derived LDEVs was characterized through RNA-seq. Abundant sRNAs were identified in LDEVs, of which 35 known sequences were tRNA and ribosome RNA (rRNA) fragments, the rest were novel predicted sequences including four microRNA-like molecules with a length of 28 nt, and higher relative abundance. Mature tRNA can give rise to smaller tRNA pieces, such as tRNA halves that originated from the 5′ part or 3′ part in bacteria (Gebetsberger and Polacek, 2013). In contrast to classic bacterial sRNAs, which are very target-specific, tRNA fragments are thought to repress translation in a manner similar to microRNAs, which often regulate multiple mRNA targets (Maute et al., 2013). Bacterial expressions of RNA molecules with secondary structures are potentially able to generate miRNA molecules that can interact with the human host mRNA during bacterial infection (Shmaryahu et al., 2014). We predicted the secondary structure of enriched sequences (sRNA45 and sRNA63) and found the similarity of tRNA. It indicates the possibility of producing miRNAs with the potential to participate in microbe-host interaction. Choi et al. (2017) found that gram-negative periodontal pathogens OMVs and gram-positive oral pathogen *Streptococcus sanguinis*-derived MVs contained a large number of msRNAs ranging in length from 15 to 45 nt. Our results differ from Choi et al. (2018) in sequence length and read. The presence of cleavage products of tRNA has been reported in *E. coli* OMVs (Ghosal et al., 2015) and *P. aeruginosa* OMVs (Koeppen et al., 2016). OMVs released by *E. coli* and *P. aeruginosa* were enriched in short RNAs (15–45 nt) (Ghosal et al., 2015; Koeppen et al., 2016). The aforementioned observations indicated that *L. plantarum* secreted msRNAs and other sRNAs into the extracellular environment *via* LDEVs during its life cycle.

In addition, microbiota-derived EVs facilitate delivery and trans-kingdom exchange of sRNAs and other biomolecules, affecting the cellular function of mammalian cells. Bacterial EVs-contained sRNAs may be transferred to host cells and function as signaling molecules between bacteria and host cells. Using the proteomics, Koeppen et al. (2016) detected a large proportion of proteins in human bronchial epithelial cells downregulated by sRNA52320 within *P. aeruginosa* OMVs. Choi et al. (2017) predicted the seed regions of msRNAs (2–8 nt of 5′ msRNA) produced by periodontal pathogens matching the 3′ UTRs of human genes related to the immune system and therefore suggested that these msRNAs potentially regulate the host genes. They pointed out that additional studies such as luciferase reporter experiments could confirm whether msRNAs would mediate the expressions of host genes (Choi et al., 2017). Unfortunately, they did not carry out associated work to validate the assumption. Combining target gene prediction analysis with proteomics data, we found that Tp53 is one of the candidate target genes for sRNA71. Luciferase reporter assays and western blot revealed that sRNA71 is specifically bound to the 3′ UTR of Tp53 to block Tp53 (Figure 4). These results allowed us to determine that Tp53 could be regarded as a potential target of *L. plantarum*-derived sRNA. Our results indicated that transfection of synthetic *L. plantarum*-secreted msRNA sequence sRNA71 strongly inhibits the expression of Tp53, suggesting that the regulation of Tp53 through this sRNA could be a potential candidate for oncotherapy. In another study, overexpressed Tp53 in a CRC model could be restored to a normalized level after consumption of *Lactobacillus rhamnosus* and *L. plantarum* (Walia et al., 2018).

It has been reported that some of probiotic microorganisms, particularly *Lactobacillus* species trigger the cellular mechanisms and signaling pathways that exhibit anti-cancer effects (Ambalam et al., 2016; Chimchang et al., 2016). Besides the direct interaction of probiotics with epithelial cells in the gastrointestinal tract, the bioactive components released by probiotics have crucial effects on microbe-host interactions. In this study, we isolated the LDEVs as one of the bioactive components from the medium of *L. plantarum* WCFS1 and investigated the molecular functions of sRNA sequences. Transfection of synthetic sRNA71 mimics resulted in the downregulation of seven proteins connected to apoptosis. We generated the hypothesis that LDEVs packaging sRNA71 could mediate host cell apoptosis, thus reflecting a novel anti-cancer component of LDEVs. Regarding the biological efficacy of EVs, physiological amounts and types of biomolecules still require more studies to investigate and verify. Behzadi et al. (2017) reported that EVs derived from *Lactobacillus rhamnosus* GG inhibited the growth of hepatic cancer cells. Whereas the key ingredient remains unclear. To sum up, sRNA has the potential to participate in cross-kingdom communication between microorganisms and the host, which possibly provides theoretical support for the anti-cancer effects of probiotics such as *L. plantarum;* therefore it is important in understanding the relationship between potential probiotics and human health.

## Materials and Methods

### Isolation and Purification of *Lactobacillus plantarum*-Derived Extracellular Vesicles

*Lactobacillus plantarum* WCFS1 was inoculated in *de* Man, Rogosa, Sharpe (MRS) broth and cultured at 37^°^C for 24 h. The isolation steps of bacterial MVs were achieved as described elsewhere (Ye et al., 2017; Yu et al., 2019) with some modifications. In brief, 500 mL bacterial culture supernatants were collected *via* centrifugation at 20,000 × *g* for 1 h at 4^°^C. Then, the supernatants were subjected to the filtration through a 0.45 μm filter, and ultracentrifugation (Optima™ XPN, Beckman, United States) at 150,000 × *g* for 2 h at 4^°^C to precipitate the vesicles. The pellets were resuspended using sterile water without RNase or PBS. The sample was then passed through a 0.22-μm filter to obtain pure LDEVs, and stored at −80^°^C until use. The protein concentration of LDEVs was measured by a BCA kit (Beyotime Biotechnology, Shanghai, China) and bovine serum albumin was used as the standard curve.

### Small RNA Sequencing

RNAs in LDEVs were extracted with the miRNeasy Serum/Plasma Kit (Qiagen, Germany) according to the manufacturer’s protocols. Total RNA samples were submitted to a DNase treatment with RNase-Free DNase Set (Qiagen) according to the manufacturer’s instructions. The composition and quality of purified RNA were analyzed using the Agilent 2100 Bioanalyzer (Agilent Technologies, Santa Clara, CA, United States) for small RNA profiles with the RNA 6000 Pico kit. RNA quantity used for library preparation is displayed in Supplementary Figure 1C. About 10 ng RNAs were then submitted to generate the sequencing library by SMARTer smRNA-Seq kit following the recommendations of the manufacturer. Sequencing libraries were amplified by 17 cycles of RCR to obtain DNA fragments corresponding to 140–200 bp. The prepared libraries were sequenced on a Hiseq platform (Illumina, San Diego, CA, United States) for 50 bp single-end reading. Raw reads were trimmed and filtered using SeqPrep^2^ and Sickle.^3^ Clean data were mapped to the *L. plantarum* genome (GenBank accession number: NC_004567.2). All of the analysis steps were as follows: Rockhopper^4^ was used for sRNA prediction and Blast software (set e value < 1e-5) combined with databases such as sRNAMap, sRNATarBase, SIPHI, Rfam were further tested for sRNA annotation. The prediction of secondary RNA structure was constructed *via* the Vienna RNA package.^5^ The quantification of the expression of the predicted sRNA sequences was performed using RSEM software.

### Reverse Transcription-Polymerase Chain Reaction

This experiment was performed to verify the presence of several selected candidates. Total RNAs in LDEVs and HEK293T cells were extracted using Trizol LS reagent following the manufacturer’s protocols (Invitrogen/Thermo Fisher Scientific, Carlsbad, CA, United States). As a result of the absence of a suitable internal reference gene in LDEVs, an additional synthetic short-chain miRNA was used as an exogenous gene for the quantification of the abundance of sRNA sequences. External control for miRNAs was used (CR100-01, Tiangen, Dalian, China). Based on the instructions of the manufacturer, 1 μmol external control corresponding to 250 μL samples was added to Trizol LS reagent before the RNA extraction. The concentration of purified RNA was determined with a spectrophotometer. About 1 μg of RNAs were reverse transcribed to cDNA using the miRcute miRNA First-Strand cDNA kit (Tiangen, Dalian, China). The abundance of each sRNA was quantified with the miRcute miRNA qPCR Detection Kit (Tiangen, Dalian, China), performed on a Real-Time PCR system (LC96, Roche, Basel, Switzerland), and determined by taking the average of three replicates. CD200-01 (Tiangen, Dalian, China) was used for the detection of external control. Otherwise, the sRNA forward primers were listed in Table 3.

**TABLE 3:** Primers used in RT-PCR.

Names	Sequences
sRNA8	5′-GGGAGTAGTAAATGGAATAGAATGGAGTAAT-3′
sRNA16	5′-AGCATAATTACGTCTCTCCGATTTCAATA-3′
sRNA26	5′-GGCACAAACTCAATTTTTAATCTTAACTCG-3′
sRNA45	5′-GTTTCCCAGTTTCCGATGCACTT-3′
sRNA63	5′-CTCAAGTTTCCTAGTTTCCGATGCAC-3′
sRNA71	5′-GTGCAAGAGCTTTCTTGTAATTTACGTG-3′
U6 snRNA	5′-GCCCCTGCGCAAGGATGAC-3′

### Predicted Human Target Genes Related to Sequencing Small RNAs in *Lactobacillus plantarum*-Derived Extracellular Vesicles

As previously mentioned, the miRanda miRNA target prediction software scanning with a pairing score cutoff of 160 was run for vesicular sRNAs and the most promising candidates. To identify the perfect matches with human mRNAs, the TargetScan was used for evaluating the complementary sequences of sRNAs aligning with human target genes. Predicted human target genes were collected and performed further bioinformatics analysis.

### Bioinformatics Analysis

GO^6^ and KEGG pathway^7^ enrichment analysis was performed on KOBAS 3.0.^8^ The term would be filtered out if its *P*-value was greater than 0.05 after the Bonferroni correction based on KOBAS (Xie et al., 2011).

### Cell Culture

HEK293T (ATCC CRL-3216) cells were cultured in DMEM (HyClone, Logan, United States), supplemented with 1% (v/v) penicillin and streptomycin solution (Sangon Biotech, Shanghai, China) and 10% (v/v) fetal bovine serum (FBS, Sijiqing Biologic, Hangzhou, China) at 37^°^C in a humidified chamber with 5% CO_2_.

### Cell Transfection

HEK293T cells were seeded in six-well plates at 1 × 10^6^ cells per well and cultured at 37^°^C for 15–16 h. Cells were switched to an antibiotic-free medium and transfected with 50 nmol sRNA71 mimics, 50 nmol negative control, 100 nmol sRNA71 inhibitor, or 100 nmol inhibitor control (Ribobio, Guangzhou, China) using Hieff Trans™ Liposomal transfection reagent (Yeasen Biotech, Shanghai, China). After 48 h of transfection, cells were collected for follow-up experiments.

The 3′ UTR sequences of Tp53 were synthesized from the genome DNA and inserted into the pGL3 luciferase reporter vector. HEK293T cells were seeded in 24-well plates at 3 × 10^4^ cells per well. After 15–16 h culture, cells with 40–50% confluency were co-transfected with 100 ng Tp53 3′ UTR firefly luciferase reporter vector and 20 ng renilla luciferase vector with 50 nmol sRNA71 mimics or 50 nmol negative control at 37^°^C for 48 h, respectively. Each group was instituted in triplicate in an independent trial. After 48 h transfection, luciferase reporter trials were performed.

### Proteomics

After 48 h of transfection of sRNA71 mimics or negative control into HEK293T cells, an equal number of cells (1 × 10^6^) were digested and harvested. Cellular proteomics was performed on a Thermo Scientific QExactive HF platform. Information on detected peptides and proteins was searched against the Uniprot database.^9^ Bioinformatics analysis of differentially expressed proteins was conducted as described above. Mean fold changes comparing sRNA71 mimics with negative control were calculated for each protein that was detected in all replicate samples and *P*-values were calculated using the Student’s *t*-test.

### Western Blot

After 48 h of transfection, HEK293T cells were lysed in RAPI buffer. As mentioned previously (Yu et al., 2017), 50 μg samples were separated by SDS-PAGE on a 5–10% Tris-HCl gel and transferred to a PVDF membrane (Millipore, Billerica, MA, United States). Following the blocking, the membranes were incubated overnight at 4^°^C with antibodies against Tp53 and GAPDH (Sangon Biotech, Shanghai, China), respectively. Next, membranes were incubated with HRP-conjugated goat anti-rabbit IgG (Sangon Biotech, Shanghai, China) for 1 h at room temperature (RT). Signal intensity was measured with an ECL kit (Share-bio, Shanghai, China) *via* ImageQuant LAS 4000 (GE Healthcare, Uppsala, Sweden).

### Luciferase Reporter Assay

Luciferase reporter assay in HEK293T cells co-transfected sRNA71 mimics or negative control, respectively, with pGL3 luciferase reporter vector containing Tp53 3′ UTR as well as renilla luciferase vector was performed using a Dual-Luciferase Reporter^®^ Assay System (E1910, Promega, United States) according to the manufacturer’s protocol. To determine the interaction between sRNA71 and Tp53 3′ UTR, a predictive binding site within the mutant vector was used instead of the wild-type reporter plasmid. Cell lysates were prepared using 100 μL of passive lysis buffer with vigorous shaking for 20 min. The experiment was independently repeated at least three times. The activity of firefly luciferase was normalized to the activity of renilla luciferase.

### Statistics

Data were expressed as the means with standard error of the mean (SEM). The analysis was administered in the software of GraphPad Prism 5.00 (GraphPad, La Jolla, CA, United States). We assessed the comparison of two groups by the Student’s *t*-test. Statistical significance was stated as: *P < 0.05, ^**^P < 0.01, ^***^P < 0.001.

## Data Availability Statement

Small RNA sequencing data has been submitted to NCBI SRA database with an accession number: PRJNA858558.

## Author Contributions

PL conceived and directed the entire project. SY performed most experiments and wrote the manuscript. PH and CZ performed the proteomics analysis. SY, ZZ, and YQ performed the bioinformatics analysis and prepared the figures. All authors read and approved the manuscript.

## Conflict of Interest

The authors declare that the research was conducted in the absence of any commercial or financial relationships that could be construed as a potential conflict of interest.

## Publisher’s Note

All claims expressed in this article are solely those of the authors and do not necessarily represent those of their affiliated organizations, or those of the publisher, the editors and the reviewers. Any product that may be evaluated in this article, or claim that may be made by its manufacturer, is not guaranteed or endorsed by the publisher.

## Footnotes

1
http://www.Pantherdb.org/

2
http://github.com/jstjohn/SeqPrep/

3
http://github.com/najoshi/sickle/

4
http://cs.wellesley.edu/~btjaden/Rockhopper/

5
http://rna.tbi.univie.ac.at/

6
http://www.geneontology.org/

7
http://www.genome.jp/kegg/

8
http://kobas.cbi.pku.edu.cn/kobas3/

9
https://www.uniprot.org/

## Abbreviations

bp: base pair
CT: cycle threshold
DEG: differentially expressed gene
DLS: dynamic light scattering
EV: extracellular vesicle
FBS: fetal bovine serum
FPKM: fragments per kilobases of exon per million fragments mapped
GO: gene ontology
KEGG: Kyoto Encyclopedia of Genes and Genomes
LDEVs: *Lactobacillus*-derived extracellular vesicles
*L. plantarum*: *Lactobacillus plantarum*
miRNA: microRNA
milRNA: microRNA-like RNA
msRNA: microRNA-size RNA
MV: membrane vesicle
MRS: *de* Man, Rogosa Sharpe
nt: nucleotide
OMV: outer membrane vesicle
*P. aeruginosa*: *Pseudomonas aeruginosa*
RIN: RNA integrity value
RISC: RNA-induced silencing complex
RNAi: RNA interference
RNA-seq: small RNA sequencing
rRNA: ribosome RNA
RT: room temperature
RT-PCR: quantitative reverse transcription polymerase chain reaction
*S. aureus*: *Staphylococcus aureus*
SEM: standard error of the mean
sRNA: small regulatory non-coding RNA
tRNA: transfer RNA
TEM: transmission electron microscope
UTR: untranslated region.
