# Supplementary material for: Biological Functions and Cross-Kingdom Host Gene Regulation of Small RNAs in Lactobacillus plantarum-Derived Extracellular Vesicles
Source: Front Microbiol. 2022 Aug 18;13:944361. doi: 10.3389/fmicb.2022.944361 (PMC9436029; doi:10.3389/fmicb.2022.944361)
Supplement: Supplementary file 2 [file Data_Sheet_1.docx]

Supplementary Material

Siran Yu^1,2^, Zhehao Zhao^2^, Yan Qiu^2^, Gang Zhou^2^, Meiyi Zhao^2^, Piliang Hao^3^, Chengqian Zhang^3^, Jiuhong Kang^1,2*^, Ping Li^2*^

^1^ Clinical and Translational Research Center of Shanghai First Maternity and Infant Hospital, School of Life Sciences and Technology, Tongji University, Shanghai 200092, PR China

^2^ Research Center for Translational Medicine at Shanghai East Hospital, School of Life Sciences and Technology, Tongji University, 1239 Siping Road, Shanghai 200092, PR China

^3^ School of Life Science and Technology, ShanghaiTech University, Shanghai 201210, PR China

*** Correspondence:**

Corresponding to Jiuhong Kang

jhkang@tongji.edu.cn

Corresponding to Ping Li

liping01@tongji.edu.cn

## Supplementary Figures


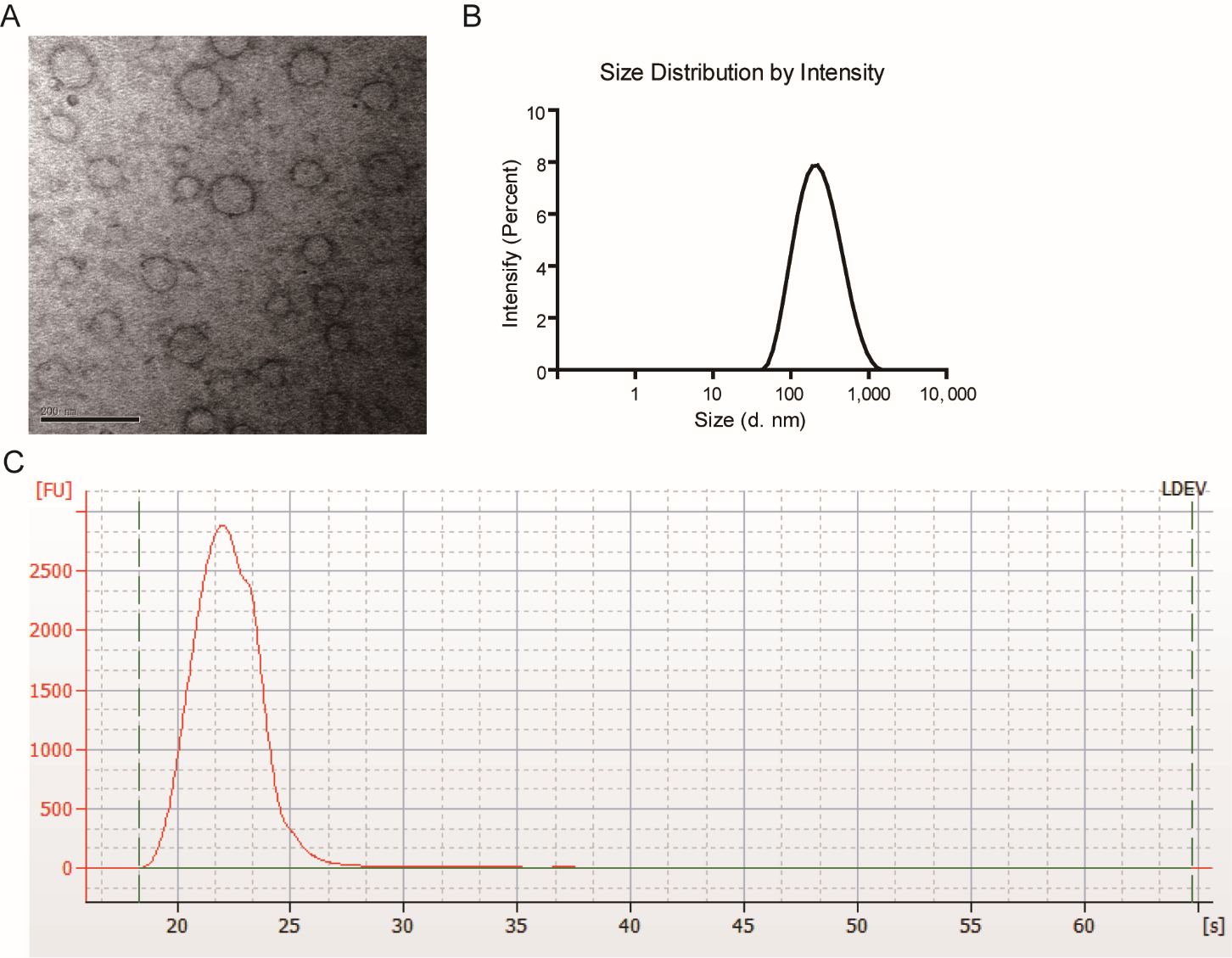


**Supplementary Figure 1.** Characterization of LDEVs produced by WCFS1. (A) The visualizations of LDEVs in transmission electron microscope (TEM) were conducted. Scale bar: 200 nm. (B) The size distributions of LDEVs by intensity were detected. (C) Total RNAs were extracted from LDEVs and analyzed using the Agilent Bioanalyzer. Graph shows RNA concentration in fluorescent units (FU) vs. nucleotide length (nt). The size range of small RNAs (sRNA) of 10-35 nt is indicated by the dotted lines.


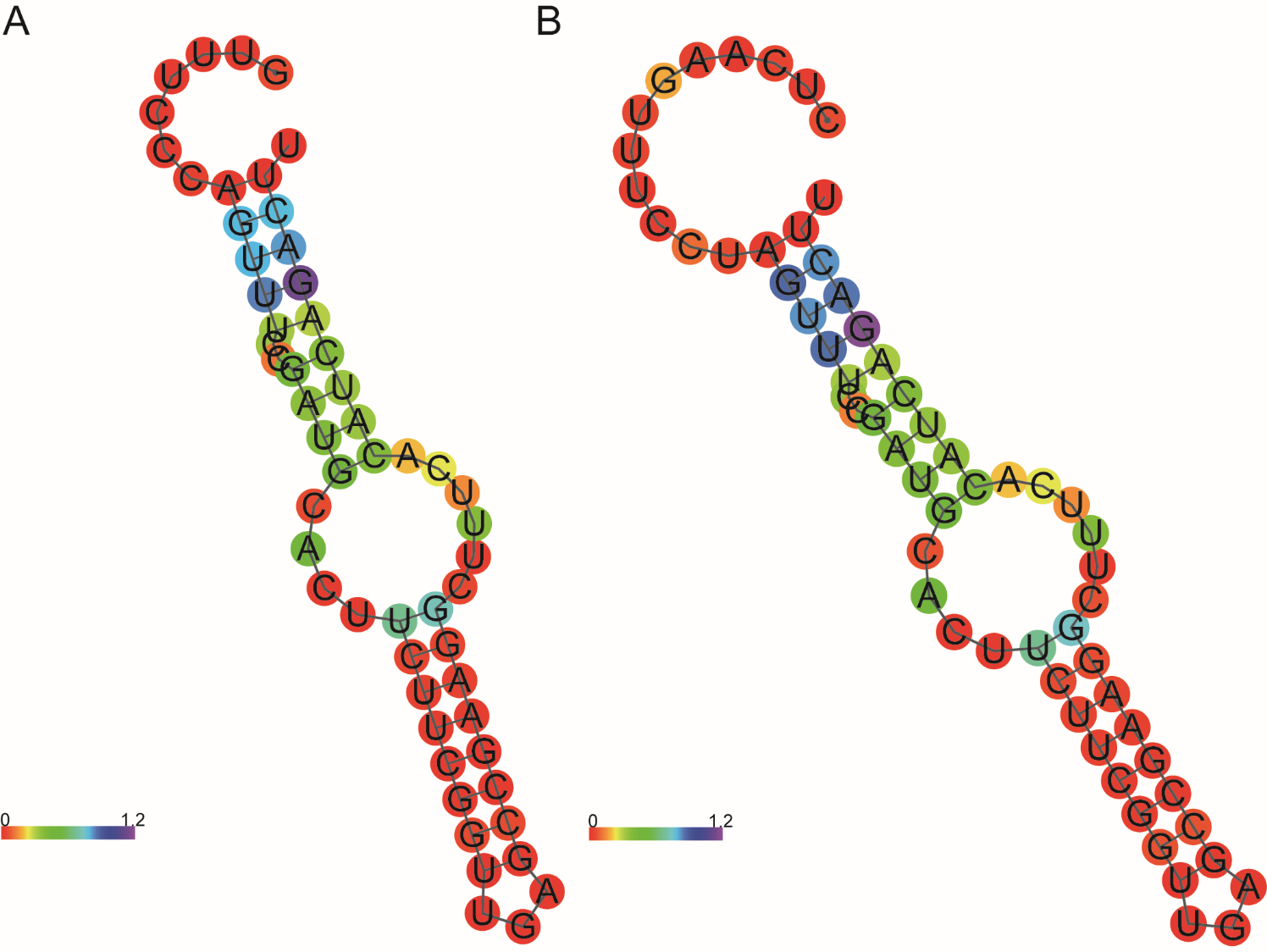


**Supplementary Figure 2.** The secondary structure of sRNA45 (A) and sRNA63 (B) was predicted via Vienna RNA package (http://rna.tbi.univie.ac.at/).


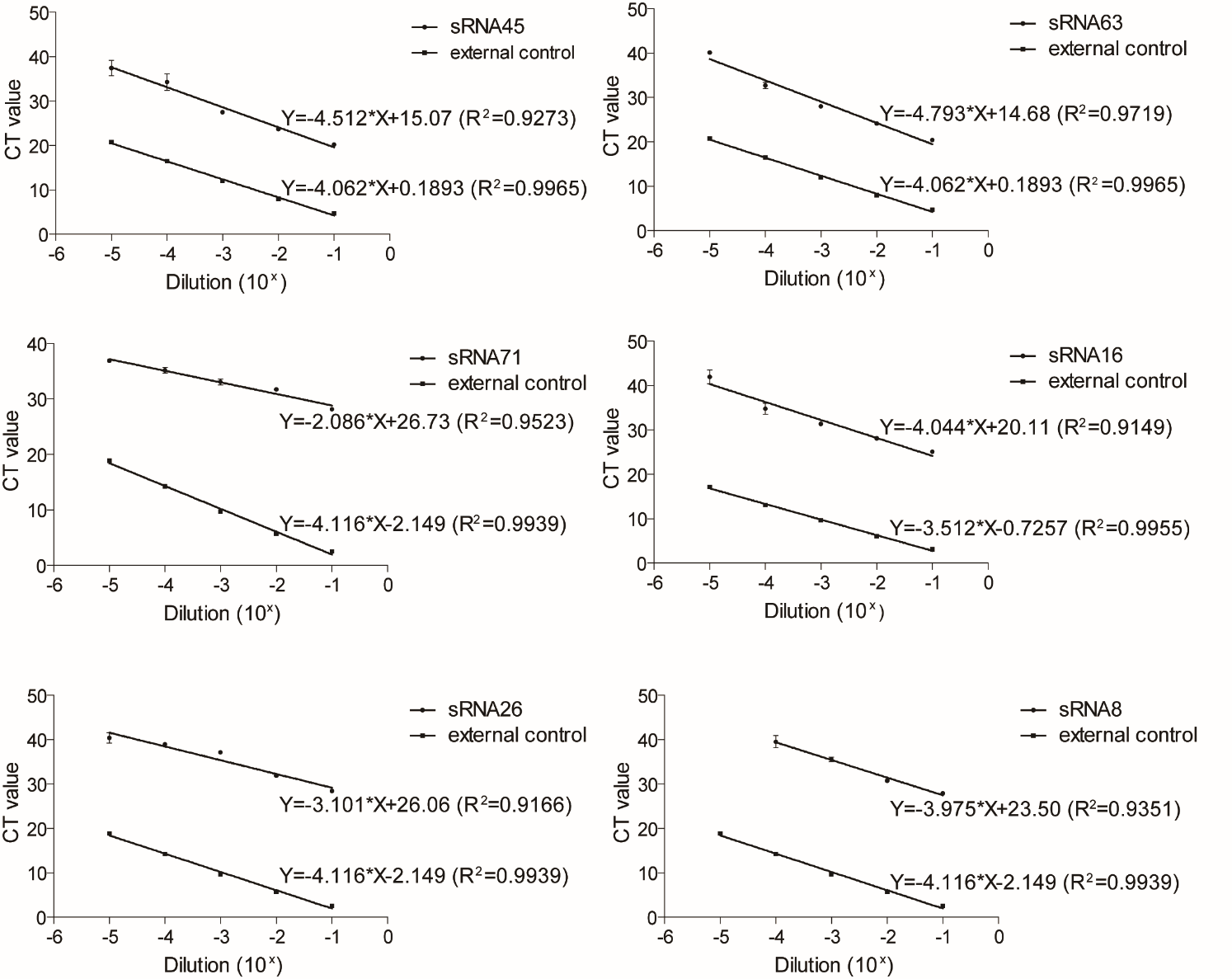


**Supplementary Figure 3.** Standard curves of each sRNA and external control for miRNA corresponding to Figure 1B. Templates in qPCR were diluted stepwise into 5 gradients by 10-fold dilution. The initial concentration of the external control is 1 pmol. All reactions were run in triplicate.


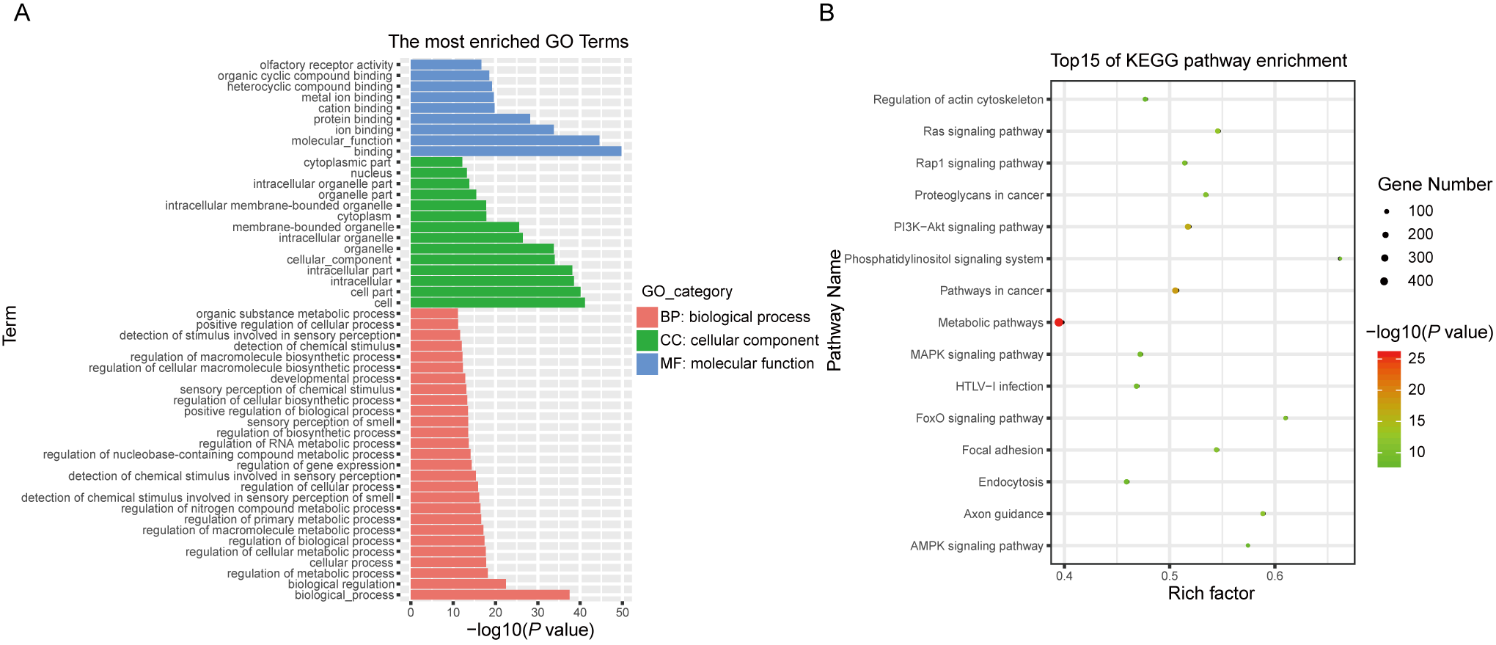


**Supplementary Figure 4.** GO (A) and KEGG pathway (B) enrichment analysis of predictive target genes of LDEVs sRNAs.


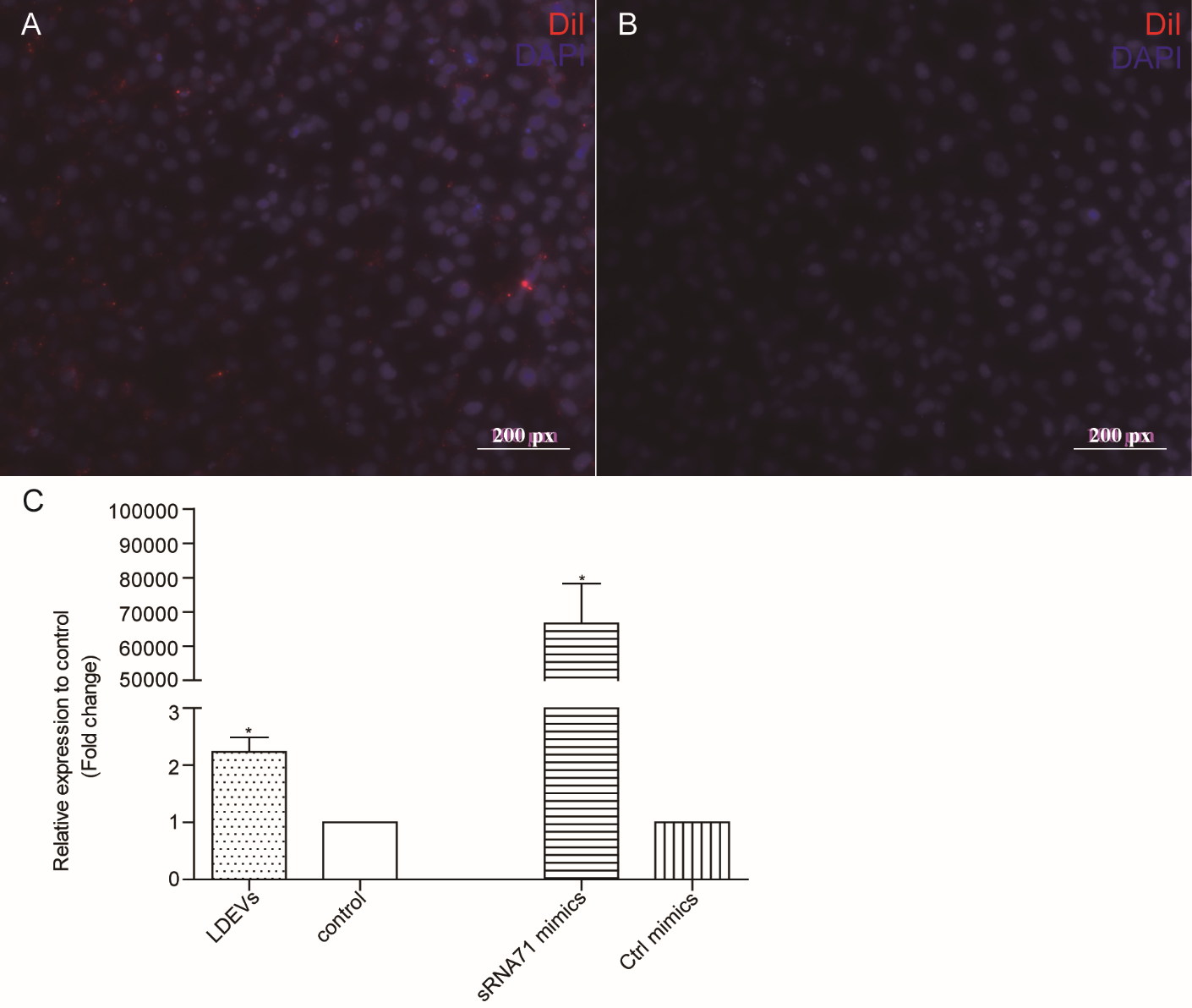


**Supplementary Figure 5.** WCFS1-derived LDEVs were uptake by mammalian cells. IEC-6 cells were co-incubated with 5 μM DiI dyed 50 μg/mL LDEVs (A) or PBS (B) at 37℃ for 4 h. Scale bar: 100 μm. (C) When reaching 80-90% confluence, 50 μg/mL LDEVs or PBS (served as control) were co-incubated with HEK293T cells at 37℃ for 6 h (left). After 48 h transfection with 50 nmol sRNA71 mimics or negative control (named ctrl mimics), HEK293T cells were collected to verify the transfection efficiency. qPCR for sRNA71 using cellular RNAs extracted from HEK293T cells with or without treatment. U6 snRNA was used as an endogenous reference for normalization. All reactions were performed in triplicate. Data are presented as mean ± SEM, **P* < 0.05 versus corresponding control.

**Materials and methods**

**Isolation and characterization of LDEVs**

EVs were extracted from culture supernatants of *Lactobacillus plantarum* WCFS1 grown overnight at 37℃ in *de* Man Rogosa Sharp (MRS) media as previously described (Yu et al., 2019; Bitto et al., 2021). The vesicle sediments from 500 mL bacterial culture were resuspended in 500 µL PBS and stored at -20℃ prior to use. The protein concentrations of LDEVs were determined by a BCA kit (Beyotime Biotechnology, Shanghai, China), a spectrophotometer (SpectraMax M5, Molecular Devices, USA) and measured at 562 nm. Bovine serum albumin was used as the standard curve. The number of EVs and their size distribution were detected by Nano-ZS 90 dynamic light scattering (zetasizer ver. 7.03, Malvern, UK) at 25℃.

Transmission electron microscopy (JEM-1230, JEOL, Japan) is used for the morphology detection. LDEVs were coated onto carbon-coated 400 mesh copper grids by placing TEM grids on top of a 10 μL droplet of LDEVs for 10 min. Samples were subsequently fixed in 2.5% (w/v) glutaraldehyde in PBS overnight and stained with 2% (w/v) uranyl acetate for 10 min. Samples were air dried and viewed using a transmission electron microscope operated at 200 kV. Each diameter was averaged more than five images under the ImageJ (<http://imagej.nih.gov/ij/index.html>/).

**Reverse transcription-polymerase chain reaction**

RNAs were extracted using Trizol LS reagent following the manufacturer’s protocols (Invitrogen/Thermo Fisher Scientific, Carlsbad, CA, USA). Due to the absence of a suitable internal reference gene in LDEVs, we used external control for miRNAs (CR100-01, Tiangen, Dalian, China) for the quantification of the abundance of predicted sRNA sequences. The concentration of RNAs in LDEVs was determined by Nanodrop. 1 µg RNAs were reverse transcribed to cDNA using the miRcute miRNA First-Strand cDNA kit (KR211, Tiangen, Dalian, China). The abundance of each sRNA was quantified with the miRcute miRNA qPCR Detection Kit (FP411, Tiangen, Dalian, China), performed on a Real-Time PCR system (LC96, Roche, Basel, Switzerland). All reactions were performed in triplicate. U6 snRNA is considered to be a reference (housekeeping) gene for normalizing the expression of sRNAs and confirming the transfection. Primer of sRNA71 is 5′-GTGCAAGAGCTTTCTTGTAATTTACGTG-3′. Primer of U6 snRNA is 5′-GCCCCTGCGCAAGGATGAC-3′.

**Cell culture, uptake, and transfection**

Rat intestinal epithelial cells (IEC-6) were purchased from the Type Culture Collection of the Chinese Academy of Sciences (Shanghai, China) and cultured in RPMI 1640 media (HyClone, Logan, USA), supplemented with 1% penicillin and streptomycin solution (Sangon Biotech, Shanghai, China) and 10% fetal bovine serum (FBS, Sijiqing Biologic, Hangzhou, China). The media were replaced with fresh liquid every 48 h. Cells were cultured in 37°C incubator with a humidified atmosphere of 5% CO_2_. HEK293T (ATCC CRL-3216) cells were cultured in DMEM (HyClone, Logan, USA), supplemented with 1% (v/v) penicillin and streptomycin solution and 10% FBS.

LDEVs were labeled with 5 μM DiI (Yeasen Biotech, Shanghai, China) for 1 h. Redundant dye was removed by ultracentrifugation at 150,000×*g* for 1 h before co-culturing with cells. IEC-6 cells were cultured overnight at 37℃ before treated with labeled LDEVs for 4 h. IEC-6 cells were washed twice by PBS. Cells were also mixed with DAPI (4’,6-diamidino-2-phenylindole) staining solution at the concentration of 20 μg/mL for another 30 min. After the incubation, cells were washed and cultures were replaced by new media. Finally, IEC-6 were observed employing fluorescence microscope (Eclipse 80i, Nikon, Japan) and captured via NIS-Elements software. All images were taken at the same exposure time and contrast ratio. The objective is 20×. The emission and excitation wavelength are 567 and 550 nm, respectively.

HEK293T cells were cultured until 80-90% confluent, and then treated with 50 μg/mL LDEVs for 6 h. As for the control, PBS instead of LDEVs was added to HEK293T. After the co-incubation, total RNAs from cells were extracted for further investigation. HEK293T cells were switched to antibiotic-free medium and transfected with 50 nmol sRNA71 mimics, or 50 nmol negative control (Ribobio, Guangzhou, China) using Hieff Trans^TM^ Liposmal transfection reagent (Yeasen Biotech, Shanghai, China). After 48 h of transfection, cells were collected for follow-up experiments.

**Luciferase assays**

The 3' UTR sequences of Tp53 was cloned into the pGL3 luciferase reporter vector using the HieffClone Plus One Step Cloning Kit. The primers used for Tp53 3' UTR cloning are 5′-CGGGGTACCCATTCTCCACTTCTTGTTCCCC-3′ and 5′-GGAAGATCTCACCCCTCAGACACACAGGTG-3′. The mutant of Tp53 3' UTR was performed by point mutant at the predicted binding site.

**Supplementary Tables**

**Supplementary Table 1 RNA Sequencing Data**

**Supplementary Table 2 sRNA71 Predicted genes**

**Supplementary Table 3 KEGG pathway enrichment analysis of sRNA71 predicted genes**

**Supplementary Table 4 Differentially expressed proteins after sRNA71 mimics transfection**

**Supplemental Reference**

Bitto, N. J., Cheng, L., Johnston, E. L., Pathirana, R., Phan, T. K., and Poon, I., et al. (2021). Staphylococcus aureus membrane vesicles contain immunostimulatory DNA, RNA and peptidoglycan that activate innate immune receptors and induce autophagy. *J Extracell Vesicles*. 10(6), e12080. doi: 10.1002/jev2.12080

Yu, S., Zhao, Z., Xu, X., Li, M., Li, P. (2019). Characterization of three different types of extracellular vesicles and their impact on bacterial growth. *Food Chem.* 272, 372-378. doi: 10.1016/j.foodchem.2018.08.059
